# Prostate cancer risk regions at 8q24 and 17q24 are differentially associated with somatic *TMPRSS2:ERG* fusion status

**DOI:** 10.1093/hmg/ddw349

**Published:** 2016-10-18

**Authors:** Manuel Luedeke, Antje E. Rinckleb, Liesel M. FitzGerald, Milan S. Geybels, Johanna Schleutker, Rosalind A. Eeles, Manuel R. Teixeira, Lisa Cannon-Albright, Elaine A. Ostrander, Steffen Weikert, Kathleen Herkommer, Tiina Wahlfors, Tapio Visakorpi, Katri A. Leinonen, Teuvo L.J. Tammela, Colin S. Cooper, Zsofia Kote-Jarai, Sandra Edwards, Chee L. Goh, Frank McCarthy, Chris Parker, Penny Flohr, Paula Paulo, Carmen Jerónimo, Rui Henrique, Hans Krause, Sven Wach, Verena Lieb, Tilman T. Rau, Walther Vogel, Rainer Kuefer, Matthias D. Hofer, Sven Perner, Mark A. Rubin, Archana M. Agarwal, Doug F. Easton, Ali Amin Al Olama, Sara Benlloch, The PRACTICAL consortium, Josef Hoegel, Janet L. Stanford, Christiane Maier

**Affiliations:** 1 Institute of Human Genetics, University of Ulm, Ulm, Germany,; 2 Department of Urology, University of Ulm, Ulm, Germany,; 3 Fred Hutchinson Cancer Research Center, Division of Public Health Science, Seattle, Washington, USA; 4 Cancer, Genetics and Immunology, Menzies Institute for Medical Research, University of Tasmania, Hobart, Tasmania, Australia,; 5 Institute of Biomedical Technology/BioMediTech, University of Tampere, Tampere, Finland,; 6 Department of Medical Biochemistry and Genetics, University of Turku, and Tyks Microbiology and Genetics, Department of Medical Genetics, Turku University Hospital, Turku, Finland,; 7 The Institute of Cancer Research, London, UK,; 8 Royal Marsden National Health Service Foundation Trust, London and Sutton, UK,; 9 Department of Genetics, Portuguese Oncology Institute, Porto, Portugal,; 10 Abel Salazar Biomedical Sciences Institute, Porto University, Porto, Portugal,; 11 Division of Genetic Epidemiology, Department of Medicine, University of Utah School of Medicine, Salt Lake City, UT, USA,; 12 George E. Wahlen Department of Veterans Affairs Medical Center, Salt Lake City, UT, USA,; 13 National Human Genome Research Institute, NIH, Bethesda, MD, USA,; 14 Department of Urology, Vivantes Humboldt Hospital, Berlin, Germany,; 15 Department of Urology, University Hospital Charité, Berlin, Germany,; 16 Department of Urology, Klinikum rechts der Isar, Technical University of Munich, Munich, Germany,; 17 Fimlab Laboratories, Tampere University Hospital, Tampere, Finland,; 18 Department of Urology, Tampere University Hospital and School of Medicine, University of Tampere, Tampere, Finland,; 19 Department of Biological Science, University of East Anglia, Norwich, UK,; 20 Department of Pathology, Portuguese Oncology Institute, Porto, Portugal,; 21 Department of Urology, Friedrich-Alexander University of Erlangen-Nürnberg, Erlangen, Germany,; 22 Institute of Pathology, Friedrich-Alexander University of Erlangen-Nürnberg, Erlangen, Germany,; 23 Institute of Pathology, University Bern, Bern Switzerland,; 24 Department of Urology, Klinik am Eichert, Göppingen, Germany,; 25 Department of Urology, Northwestern University Feinberg School of Medicine, Chicago, IL, USA,; 26 Pathology of the University Medical Center Schleswig-Holstein, Campus Luebeck and the Research Center Borstel, Leibniz Center for Medicine and Biosciences, Luebeck and Borstel, Germany,; 27 Department of Pathology and Laboratory Medicine, Weill Medical College of Cornell University, New York, NY, USA,; 28 University of Utah/ARUP Laboratories, Salt Lake City, UT, USA,; 29 Centre for Cancer Genetics Epidemiology, Department of Oncology, University of Cambridge, Cambridge, UK; 30 Department of Epidemiology, School of Public Health, University of Washington, Seattle, Washington, USA

## Abstract

Molecular and epidemiological differences have been described between *TMPRSS2:ERG* fusion-positive and fusion-negative prostate cancer (PrCa). Assuming two molecularly distinct subtypes, we have examined 27 common PrCa risk variants, previously identified in genome-wide association studies, for subtype specific associations in a total of 1221 *TMPRSS2:ERG* phenotyped PrCa cases. In meta-analyses of a discovery set of 552 cases with *TMPRSS2:ERG* data and 7650 unaffected men from five centers we have found support for the hypothesis that several common risk variants are associated with one particular subtype rather than with PrCa in general. Risk variants were analyzed in case-case comparisons (296 *TMPRSS2:ERG* fusion-positive versus 256 fusion-negative cases) and an independent set of 669 cases with *TMPRSS2:ERG* data was established to replicate the top five candidates. Significant differences (*P* < 0.00185) between the two subtypes were observed for rs16901979 (8q24) and rs1859962 (17q24), which were enriched in *TMPRSS2:ERG* fusion-negative (OR = 0.53, *P* = 0.0007) and *TMPRSS2:ERG* fusion-positive PrCa (OR = 1.30, *P* = 0.0016), respectively. Expression quantitative trait locus analysis was performed to investigate mechanistic links between risk variants, fusion status and target gene mRNA levels. For rs1859962 at 17q24, genotype dependent expression was observed for the candidate target gene *SOX9* in *TMPRSS2:ERG* fusion-positive PrCa, which was not evident in *TMPRSS2:ERG* negative tumors. The present study established evidence for the first two common PrCa risk variants differentially associated with *TMPRSS2:ERG* fusion status. *TMPRSS2:ERG* phenotyping of larger studies is required to determine comprehensive sets of variants with subtype-specific roles in PrCa.

## Introduction

Prostate cancer (PrCa) is a complex disease with a considerable degree of heritability involved in its etiology (1). Although high-risk gene discovery has proven difficult against a background of disease and locus heterogeneity, genome-wide association studies (GWAS) and substantial validation efforts have identified more than 100 common variants with weak to moderate contributions to PrCa risk (2–11). These common risk variants are postulated to explain about 33% of the familial risk of PrCa (12).

Somatically, PrCa can be classified into two major molecular subtypes, where the presence or absence of oncogenic E-twenty-six (ETS) gene fusions is the crucial distinctive feature. ETS rearrangements are present in ∼50% of PrCa tissues (13) and their occurrence is considered an early event in PrCa tumorigenesis (14). In over 90% of ETS fusion-positive cases, the fusion partners are the androgen-regulated gene *TMPRSS2* (transmembrane protease, serine 2), which is highly expressed in the prostate, and the oncogene *ERG* (v-ets avian erythroblastosis virus E26 oncogene homolog), both located on the long arm of chromosome 21 (13).

Since the discovery of ETS gene fusions in PrCa multiple studies have provided evidence for the molecular and epidemiological distinctness of *TMPRSS2:ERG* fusion-positive and negative tumors. Epigenetic profiling has revealed distinct DNA methylation patterns for *TMPRSS2:ERG* fusion-positive and negative PrCa tissues (15–17) and analyses of benign and tumor tissues suggest that hypermethylation is more pronounced in *TMPRSS2:ERG* fusion-negative PrCa compared with *TMPRSS2:ERG* fusion-positive tumors, which mostly show moderately elevated DNA methylation (16,17). During tumor evolution of fusion-positive PrCa interdependent complex rearrangements (chromoplexy) occur at transcriptionally active—predominantly androgen regulated—loci of multiple chromosomes, while fusion-negative tumors tend to undergo single fatal genetic restructuring events (chromothripsis) (18,19). In addition to tumor architecture, differences in clinical and epidemiological characteristics have also been investigated for *TMPRSS2:ERG* positive and negative PrCa. Although a correlation of more aggressive PrCa with fusion status has not been reported consistently (20), *TMPRSS2:ERG* fusions have been found more frequently in early onset PrCa (21,22). Interestingly, the frequency of *TMPRSS2:ERG* fusions varies among ethnicities with the highest prevalence in cases of European ancestry (23). Moreover, individual physiologic and metabolic factors appear to have different risk modifying effects for *TMPRSS2:ERG* positive and negative PrCa (24,25).

Based on their distinctness, we hypothesized that there may also be differences between *TMPRSS2:ERG* fusion-positive and negative PrCa at the underlying germline level. Within the framework of the PRACTICAL consortium, we have investigated the first confirmed 27 common risk variants, which were identified in PrCa GWAS studies (4), for fusion-specific associations. For this purpose, we have analyzed a set of 296 *TMPRSS2:ERG* positive and 256 negative cases for differences in variant allele frequencies between these subtypes, and additionally, both subgroups were compared with controls without PrCa (n = 7650). The five top-ranked candidate variants were then genotyped in an independent sample of 669 PrCa cases with known *TMPRSS2:ERG* status for replication purposes. For the highlighted risk regions, we considered mRNA expression analysis of candidate target genes in fusion-positive and negative tumor tissues, to investigate the mechanistic interplay between the somatic *TMPRSS2:ERG* phenotype and the germline genotype of associated risk variants.

## Results

### Quality control and eligibility of the hypothesis generating discovery dataset

The five participating studies (FHCRC, IPO-PORTO, TAMPERE, UKGPCS and ULM) consisted of a total number of 7650 controls and 8681 cases previously genotyped for the iCOGS study (7). From the available iCOGS array genotype data, we selected 27 variants, representing the initial set of confirmed common PrCa risk variants, for analyzing potential associations with *TMPRSS2:ERG* fusion status. None of these variants showed deviation from Hardy-Weinberg equilibrium (threshold *P* = 0.001) in any of the study populations.

A subgroup of 552 cases genotyped as part of the iCOGS dataset was somatically phenotyped for the *TMPRSS2:ERG* gene fusion with a mean *TMPRSS2:ERG* positive frequency of 54% (range 44–60%) across the study groups (Table 1). Since the patients with *TMPRSS2:ERG* data represented only a fraction of the total cases from each collaborating center, two validity issues were considered in supplemental analyses. First, we checked for potential bias that may have occurred in the course of subsampling tumor materials. For this question, risk allele frequencies for all 27 loci were compared between somatically phenotyped cases (n = 552) and the 8129 non-phenotyped cases from the same contributing sites by Mantel-Haenszel analysis (under a fixed-effects model). Using this approach, sampling bias was observed for one variant (rs7127900 at 11p15.5; *P* = 0.0056), which was consequently omitted from further analyses. For all other 26 variants, the phenotyped cases did not differ significantly from the untyped cases (*P* > 0.12; data not shown), and were therefore considered as representative of the entire case groups. Of note, no significant cancer-related sampling bias was indicated by clinical features, such as tumor stage (organ confined vs. advanced: *P* = 0.11) or tumor grade (Gleason Score ≤ 7 versus >7: *P* = 0.39).

**Table 1.** ** **Distribution of PrCa cases based on study centers and *TMPRSS2:ERG* (*T2E*) fusion status

	*Study*	*Total number of cases with T2E data*	*T2E positive cases*	*T2E negative cases*	*T2E positive frequency*
Discovery sample	FHCRC I	174	91	83	0.52
	IPO-PORTO I	18	8	10	0.44
	TAMPERE	174	105	69	0.60
	UKGPCS	129	58	71	0.45
	ULM I	57	34	23	0.60
	Subtotal	552	296	256	0.54

Replication sample	FHCRC II	218	133	85	0.61
	IPO-PORTO II	146	79	67	0.54
	ULM II	107	65	42	0.61
	BERLIN	198	111	87	0.56
	Subtotal	669	388	281	0.58

	Total	1221	684	537	0.56

A second issue of validity was examined with respect to the relatively small effect sizes of common risk variants, questioning if subsampling may reduce our power for detecting any associations with overall risk of PrCa, or risk in the two PrCa subgroups stratified by fusion status. Using all 881 unselected cases in case-control comparisons, 20 out of the 26 ‘bona fide’ PrCa risk variants replicated at a threshold of *P* < 0.00185 (corresponding to Bonferroni correction for the 27 variants included in this study). However, after reduction to 552 *TMPRSS2:ERG* phenotyped cases, only six variants remained significantly associated with PrCa risk (Supplementary Material, Table S1), suggesting that larger sample sizes are likely required for the remaining variants to achieve adequate power for subset analyses.

### Case-control comparisons according to TMPRSS2:ERG fusion status suggest common risk variants with subtype preference

**Figure 1.**
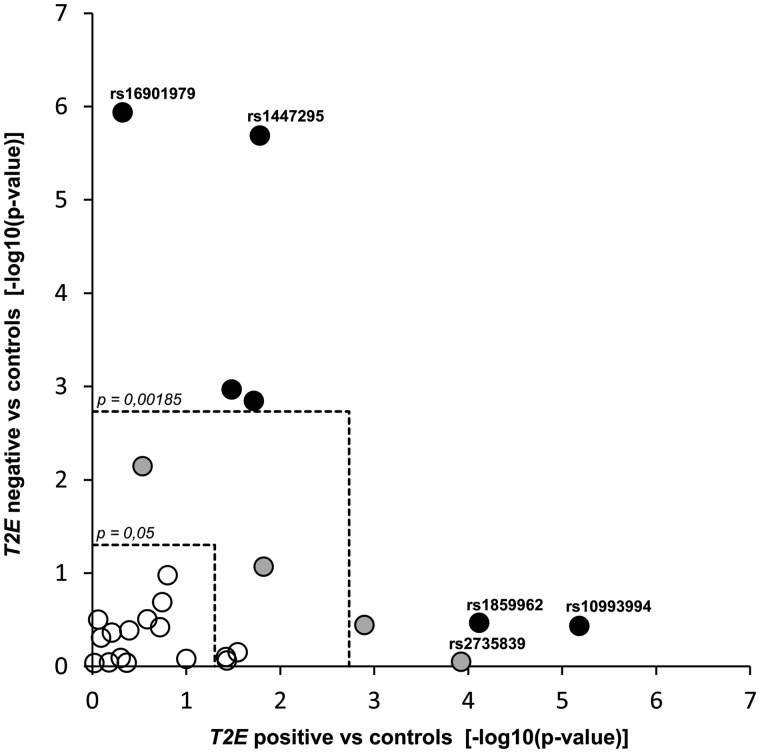
Mantel-Haenszel analysis showing associations between common PrCa risk variants and *TMPRSS2:ERG* (*T2E*) positive (x-axis) and *T2E* negative cases (y-axis) compared with controls. Analyses included n = 296 *T2E* fusion-positive and n = 256 *T2E* fusion-negative PrCa cases, which were separately compared with n = 7650 controls. Threshold lines correspond to nominal significance (*P* = 0.05, inner dashed square) and study significance adjusted for 27 variants (*P* = 0.00185, outer dashed square). Circles are colored based on separate analyses, where the variants were pre-checked for overall association with PrCa risk in all phenotyped cases (n = 552) versus all controls (Open circles: *P* > 0.05; gray: *P* < 0.05; black: *P* < 0.00185; Supplementary Material, Table S1). The majority of common risk variants was not associated with PrCa risk in the *T2E* phenotyped sample as compared with controls, and these remain unrelated to molecular subtype. Candidates significantly associated with PrCa risk showed stronger or unique associations for either *T2E* positive or negative PrCa. No variant was significantly associated with both subtypes. The highest ranked candidate variants, which were later genotyped in a replication dataset, are annotated with variant rs ID numbers.

Potential subtype preference for the 26 candidate variants were examined by comparing the groups of *TMPRSS2:ERG* fusion-positive (n = 296) and fusion-negative cases (n = 256) to the 7650 controls (Supplementary Material, Table S1). The six risk variants that were associated with PrCa by comparing all 552 *TMPRSS2:ERG* phenotyped cases to controls and two additional variants appeared to be associated with either *TMPRSS2:ERG* fusion-positive or fusion-negative PrCa. Four variants were associated with *TMPRSS2:ERG* positive PrCa and four with *TMPRSS2:ERG* negative PrCa at the study-wide significance threshold of *P* = 0.00185 (Supplementary Material, Table S1). The strongest associations were observed between *TMPRSS2:ERG* negative PrCa and two independent risk variants at 8q24 (rs16901979, region 2 (R2), *P* = 1.2 × 10^−^^6^; and rs1447295, region 1 (R1), *P* = 2.0 × 10^−^^6^). Figure 1 displays all variants with their significance in the total phenotyped sample (color codes), in fusion-positive cases (x-axis) and in fusion-negative cases (y-axis) as compared with controls, respectively. Variants with stronger effect sizes (as ranked in Supplementary Material, Table S1) tended towards having associations with one somatic subtype, but not with both. This view supports the hypothesis that subtype specific common germline variants most likely exist.

### TMPRSS2:ERG fusion-positive PrCa versus fusion-negative PrCa revealed differentially associated loci at 8q24 and 17q24

We then assessed differences in risk allele frequencies between the two somatic subtypes by case-case comparisons of the 296 *TMPRSS2:ERG* positive and the 256 *TMPRSS2:ERG* negative cases. Mantel-Haenszel results for all variants are presented in Supplementary Material, Table S2. No strong evidence for heterogeneity between study centers was observed. Nominally significant differences between *TMPRSS2:ERG* positive and negative cases were present for four variants. These include three variants with a higher risk allele frequency in *TMPRSS2:ERG* positive cases: rs10993994 at 10q11 (*P* = 0.015), rs2735839 at 19q13 (*P* = 0.0035) and rs1859962 at 17q24 (*P* = 0.038). One risk variant at 8q24 (rs16901979, R2) was more frequent in fusion-negative cases (*P* = 0.021). The second variant at 8q24 (rs1447295, R1), which was strongly associated with *TMPRSS2:ERG* negative PrCa when compared with controls, showed a similar tendency towards enrichment of the risk allele in *TMPRSS2:ERG* negative versus positive PrCa, although this result was not significant (*P* = 0.0891).

To substantiate findings of differential associations from the hypothesis generating dataset, an additional 669 independent cases with *TMPRSS2:ERG* phenotype data were used for case-case comparisons. The patients from four different study centers, FHCRC, IPO-PORTO, ULM and BERLIN, contained similar proportions of *TMPRSS2:ERG* positive (n = 388; 58%) and negative cases (n = 281; 42%) as the initial discovery set (Table 1). For genotyping, the top five candidate variants were selected based on results from the initial *TMPRSS2:ERG* subgroup case-control analyses (Supplementary Material, Table S1) and from case-case comparisons as ranked in Supplementary Material, Table S2. In this independent patient dataset, case-case comparisons found nominally significant associations between three variants and *TMPRSS2:ERG* subtype, each in the same direction as observed in the discovery sample (Table 2 and Supplementary Material, Fig. S1). The strongest associations were seen for rs1447295 (8q24, R1; *P* = 0.0085) and rs16901979 (8q24, R2; *P* = 0.012), where the risk alleles were enriched in *TMPRSS2:ERG* negative cases, and rs1859962 (17q24), where the risk allele was enriched in *TMPRSS2:ERG* positive cases (*P* = 0.018). The results for variants rs10993994 (10q11) and rs2735839 (19q13) were not confirmed in the independent dataset. In combined analyses of all 1221 phenotyped cases from the discovery and the replication sets, rs16901979 (8q24 R2; *P* = 0.0007) and rs1859962 (17q24; *P* = 0.0016) reached study significance (*P* < 0.00185), while rs1447295 (8q24 R1; *P* = 0.0025) was close to this threshold.

**Table 2.** ** **Association results for the top five PrCa risk variants and *TMPRSS2:ERG* fusion status in the discovery sample, replication sample and both samples combined calculated by Mantel-Haenszel analyses^a^

Variant	Discovery sample		Replication sample		Combined analysis	
	OR^b^ [95% CI]	*P*-value	OR^b^ [95% CI]	*P*-value	OR^b^ [95% CI]	*P*-value
rs16901979	0.53 [0.31–0.91]	0.0214	0.53 [0.33–0.87]	0.0121	0.53 [0.37–0.76]	0.0007
rs1447295	0.76 [0.56–1.04]	0.0891	0.63 [0.44–0.89]	0.0085	0.70 [0.55–0.88]	0.0025
rs10993994	1.35 [1.06–1.72]	0.0151	1.10 [0.89–1.37]	0.3789	1.21 [1.03–1.42]	0.0226
rs2735839	1.73 [1.20–2.51]	0.0035	1.03 [0.76–1.39]	0.8650	1.27 [1.00–1.59]	0.0455
rs1859962	1.29 [1.01–1.64]	0.0375	1.30 [1.05–1.62]	0.0178	1.30 [1.10–1.52]	0.0016

^a^Sample numbers are given in Table 1, corresponding forest plots and study heterogeneity are shown in Supplementary Material, Figure S1.

^b^ORs < 1 imply an overrepresentation of PrCa risk alleles in *TMPRSS2:ERG* fusion-negative cases, whereas ORs above 1 indicate an overrepresentation in *TMPRSS2:ERG* fusion-positive cases.

The main analysis addressed allelic association only, regardless of genetic models on genotypes. However, the crude *TMPRSS2:ERG* fusion frequencies in cases displayed by genotypes revealed additive effect tendencies (Supplementary Material, Fig. S2). This observation is particularly striking for the 8q24 variants associated with fusion-negative PrCa, where homozygous carriers showed a *TMPRSS2:ERG* frequency of only one-third, in contrast to the overall frequency of 56%.

### Potential confounders

As previous studies have reported that *TMPRSS2:ERG* fusions have a higher prevalence in cases with early onset PrCa, we investigated whether age at diagnosis was potentially confounding the observed results of our confirmed variants, rs16901979 and rs1859962. In our dataset, age at diagnosis was significantly associated with *TMPRSS2:ERG* status (crude OR = 0.96 per year, *P* = 4.7 × 10^−^^5^; Supplementary Material, Table S3). Of note, the two variants at 8q24 and 17q24 were not associated with age at diagnosis (rs16901979: *P* = 0.38; rs1859962: *P* = 0.88). In multivariable logistic regression analyses, the association between age at diagnosis and *TMPRSS2:ERG* status did not change when adjusted for each variant (Supplementary Material, Table S3). Similarly, the associations between fusion status and the variants rs16901979 and rs1859962 were not modified when age at diagnosis was included in the model. In conclusion, age at diagnosis and the risk variants, 16901979 and rs1859962, are independent predictors of *TMPRSS2:ERG* status.

As a potential technical confounder, we considered differences in *TMPRSS2:ERG* detection methods among study samples. The inclusion of different *TMPRSS2:ERG* detection methods (i.e. fluorescence *in situ* hybridization (FISH) or RT-PCR) as a covariable, in addition to study center, revealed little differences in the associations between *TMPRSS2:ERG* subtype and the variants, rs16901979 and rs1859962 (Supplementary Material, Table S3).

### EQTL analyses suggest subtype and genotype specific SOX9 mRNA expression at 17q24

The variants rs16901979 (8q24) and rs1859962 (17q24) are both located within gene deserts, where long-range interactions have been assumed between transcriptional regulatory elements and distant genes, such as *MYC* at 8q24 and *SOX9* at 17q24. Expression levels of target genes could provide useful insights into how germline risk variants exert their effects, in particular in tumor subtypes according to *TMPRSS2:ERG* fusion status. From three cohorts, 262 fresh-frozen tumor samples were available for expression quantitative trait locus (eQTL) analysis and 70 matched sample pairs for comparing gene expression between tumor and adjacent benign tissue. With regards to the 8q24 variant, rs16901979, the rarity of the risk allele (frequency 0.04 in *TMPRSS2:ERG* fusion-positive and 0.07 in fusion-negative cases) resulted in insufficient genotype counts for generating adequate eQTL categories in the two subtypes, thus, this locus could not be investigated. For the 17q24 locus, we chose *SOX9* as a candidate target gene based on previous studies (26) and assessed whether the observed differential association between rs1859962 and *TMPRSS2:ERG* status is reflected in subtype- and genotype-specific mRNA expression levels.

**Figure 2.**
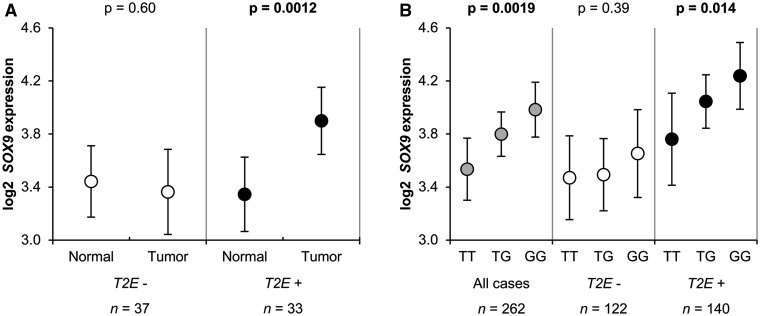
Expression levels of *SOX9* according to *TMPRSS2:ERG* fusion status in adjacent benign and matched tumor tissues **(A)** and in eQTL analyses of rs1859962 **(B)**. Mean values of log2 expression levels are presented with corresponding 95% CI. Significant *P*-values are in bold-type. (A) *SOX9* expression levels in pairs of 70 tumor and adjacent benign tissues for *TMPRSS2:ERG* fusion-negative (open circles) and fusion-positive cases (black circles). *P*-values are derived from paired *t*-tests. (B) *SOX9* expression levels according to rs1859962 genotype for all tumor samples (n = 262; gray circles), *TMPRSS2:ERG* fusion-negative samples (n = 22; open circles) and fusion-positive samples (n = 140; black circles). *P*-values correspond to the association between risk alleles and expression levels in a linear regression model.

Comparison of adjacent benign and tumor tissue revealed a significant increase in *SOX9* mRNA expression in *TMPRSS2:ERG* fusion-positive tumors (*P* = 0.0012), while the expression of *SOX9* in fusion-negative tumors resembled that of benign tissue (*P* = 0.60, Fig. 2A). Regarding the hypothesized eQTL manifestation of rs1859962 (Fig. 2B), linear regression analysis showed a significant association between *SOX9* mRNA levels and the presence of the risk allele G (effect per G allele = 0.21, *P* = 0.0019). When split by fusion status, the genotype dependency was evident in the *TMPRSS2:ERG* positive subset (effect per G allele = 0.23, *P* = 0.014). No significant association was observed in *TMPRSS2:ERG* negative tumors (effect per G allele = 0.09, *P* = 0.39). To further investigate, whether the correlation structure between rs1859962 and *SOX9* mRNA levels statistically differ between *TMPRSS2:ERG* fusion-positive and negative tumors, we added an interaction term for genotype and *TMPRSS2:ERG* fusion status to the linear regression model with these two main factors. Though underpowered, this extended model demonstrated a significant impact of rs1859962 genotype (*P* = 0.021) and fusion status (*P* = 0.036) on *SOX9* mRNA levels, but could not formally prove their interrelationship (*P* = 0.31).

## Discussion

Since the discovery of ETS gene rearrangements in PrCa, numerous efforts have sought to determine whether fusion-positive and fusion-negative tumors differ with respect to clinical significance, pathology and tumorigenesis itself. While comprehensive analyses of genomic and epigenomic alterations provide supportive evidence for distinct molecular mechanisms in the pathogenesis of fusion-positive and negative tumors (15,16,18), little is known to what extent these molecular subtypes are linked to the apparent heritable background of PrCa. Nevertheless, several previous reports have supported the hypothesis of genetically distinct tumor entities. In familial PrCa pedigrees, we have observed that relatives are more likely to share the same *TMPRSS2:ERG* subtype (27), and have found rare variants in DNA repair genes to be associated with fusion status (28). Intriguingly, after the recent identification of the hereditary PrCa gene *HOXB13* (29), in-depth pathology examination subsequently revealed subtype specific predisposition, as 83% of *HOXB13* germline mutation carriers had *TMPRSS2:ERG* negative tumors (30). With respect to common risk-modifying variants, the Physicians’ Health Study (PHS) and Health Professionals Follow-up Study (HPFS) recently examined 39 variants for subtype preference in a cohort of 227 fusion-positive and 260 negative cases (31). The authors found nominally significant associations between *TMPRSS2:ERG* fusion status and PrCa risk variants at 4q24, 5p15, 8p21, 17q24, 19q13 and 22q13. Although not withstanding correction for multiple testing, these six variants exceeded the number of associations expected by chance. In this study, consisting of a large sample of cases with *TMPRSS2:ERG* fusion data, we have substantiated the hypothesis that common risk variants are involved in particular molecular subtypes of PrCa, rather than in PrCa risk in general, and have found significant evidence that variants at 8q24 and 17q24 are differentially associated with *TMPRSS2:ERG* fusion status.

To date, associations between common risk variants and *TMPRSS2:ERG* subtypes have been investigated by the PHS/HPFS study (31) and our present work, resulting in more than 1700 PrCa cases with somatic fusion status. As these two studies used different sets of candidate SNPs, several interesting loci cannot be checked for independent confirmation between the studies, such as 5p15 (rs12653946), 19q13 (rs11672691) and 22q13 (rs11704416), which were associated with nominal significance in the PHS/HPFS dataset, but were not genotyped directly or by a proxy SNP in our study. Two further findings in the PHS/HPFS cohorts, 4q24 (rs7679673) and 8p21 (rs1512268), were genotyped in the discovery dataset of the present work, but no significant associations were observed (*P* = 0.86 and 0.45, respectively). Notably, rs1859962 at 17q24 was included in both studies, and was identified in the PHS/HPFS dataset to be nominally associated with *TMPRSS2:ERG* fusion-positive PrCa (OR = 1.32; *P* = 0.04). We observed a similar association in both of our independent datasets (discovery: OR = 1.29; *P* = 0.04 and replication OR = 1.30; *P* = 0.02) with a study-wide significance in our combined analysis (OR = 1.30; *P* = 0.0016), providing strong evidence that the 17q24 variant is preferentially associated with *TMPRSS2:ERG* fusion-positive PrCa risk. Variant rs16901979 at 8q24 was found to be associated with *TMPRSS2:ERG* negative PrCa, in both the discovery and replication datasets in our study (OR = 0.53; *P* = 0.02 and OR = 0.53, *P* = 0.01, respectively; *P* = 0.0007 combined); however this was not the case in the PHS/HPFS cohorts (OR = 0.78; *P* = 0.48). Variant rs16901979 maps to the known 8q24 PrCa risk region 2, where a variant, rs1016343, with a more frequent risk allele was genotyped in the PHS/HPFS cohort. This variant shows linkage disequilibrium to rs16901979 (*r*^2^ = 0.11; D’ = 1) and was over-represented in the PHS/HPFS *TMPRSS2:ERG* negative PrCa cases (OR = 0.75) with borderline significance (*P* = 0.06). Also of interest was the fact that the risk alleles in the independent 8q24 risk regions 3 (rs6983267, OR = 0.85, *P* = 0.19; PHS/HPFS study) and 1 (rs1447295, OR = 0.70, *P* = 0.0025; present study) were also over-represented in *TMPRSS2:ERG* negative PrCa, although with different levels of significance. In summary, the consistent tendency of multiple 8q24 risk loci to be over-represented in *TMPRSS2:ERG* fusion-negative PrCa is intriguing, and requires the study of larger cohorts to confirm or disprove the involvement of 8q24 in the fusion-negative subtype.

The association found between *TMPRSS2:ERG* positive PrCa and rs1859962 at 17q24 suggests a molecular mechanism linking the risk region to the ERG pathway. For eQTL analysis, we considered *SOX9* (*SRY* (sex determining region Y)-box 9), which is located in relatively close proximity (1 Mb) to the rs1859962 risk variant. *SOX9* acts as a transcription factor in the development of prostate epithelia and its over-expression evidently plays a role in PrCa tumorigenesis (32,33). Long-range interactions between *SOX9* and variants in LD with rs1859962 have been proposed previously in (26). *SOX9* has also been identified as a downstream target of ERG (34) and a recent large histopathological study found a strong correlation between positive ERG status and moderate and high levels of *SOX9* in PrCa tumor tissues (35). In line with *SOX9* being a downstream target of ERG, we observed *SOX9* over-expression only in fusion-positive tumors, while fusion-negative tumors have transcript levels similar to adjacent benign tissue. Remarkably, eQTL analysis stratified by fusion type demonstrated a positive correlation between *SOX9* gene expression and the rs1859962 risk allele in *TMPRSS2:ERG* positive tumor tissue. In contrast, this correlation was not evident in the *TMPRSS2:ERG* negative subset. Of note, for normal prostate tissue, where ERG should not be overexpressed, no eQTL evidence between rs1859962 and *SOX9* (*P* = 0.51) was retrieved from the GTex portal (www.http://www.gtexportal.org) (36). Taken together, these results suggest that germline risk alleles at 17q24 promote *ERG-*mediated changes in *SOX9* expression only in *TMPRSS2:ERG* fusion-positive tumors, and the synergistic effect of these factors—risk variants and *TMPRSS2:ERG* fusion—render advantages to precursor cells in tumor formation.

Recent independent studies have found that *TMPRSS2:ERG* positive tumors are more frequent in patients with an earlier age at diagnosis of PrCa (21,22). The association with age at diagnosis was also present in our study population. Several explanations for the higher incidence of *TMPRSS2:ERG* fusions in early onset patients have been proposed, including a crucial role of higher androgen levels at younger ages (21), as well as the notion that *TMPRSS2:ERG* positive tumors may develop faster leading to earlier clinical manifestation, as compared with fusion-negative PrCa (22,37). The hypothesis that specific germline variants may predispose the development of early onset *TMPRSS2:ERG*-dependent PrCa is intriguing. Of note, the risk variant rs1859962 at 17q24 has been implicated in early onset PrCa previously (38). However, regression based analyses of the present study population revealed that age at diagnosis and rs1859962 were both associated with *TMPRSS2:ERG* fusion status, but were independent of each other.

With *TMPRSS2:ERG* status as the main study focus, concerns arose as to whether different detection methods used by study groups could have biased results. Each method, i.e. FISH for formalin fixed paraffin embedded (FFPE) tissue or quantitative real-time PCR of RNA from fresh-frozen tissue, has its own spectrum of false-positive and false-negative outcomes. In particular, while the FISH break apart assay manages to detect almost every rearrangement involving *TMPRSS2* and *ERG*, including those which do not lead to a functional *TMPRSS2:ERG* isoform (over-estimation of relevant *TMPRSS2:ERG*), qPCR detection of the most prevalent *TMPRSS2:ERG* transcript may misclassify tumors harboring only rare *TMPRSS2:ERG* isoforms (underestimation of relevant *TMPRSS2:ERG*). In addition, FISH on tissue micro arrays may miss *TMPRSS2:ERG* positive tumor foci, due to the limited area of analyzed tumor tissue, while qPCR on macro-dissected fresh-frozen tumor tissue could enable a more comprehensive evaluation. We believe, however, that the different detection methods have had little effect on the results of our study. First, the *TMPRSS2:ERG* fusion frequencies among individual studies were similar to each other and the meta-analyses of the present samples revealed little evidence for heterogeneity. Second, adjustment for the detection method in multivariable regression analyses had almost no impact on the observed associations between *TMPRSS2:ERG* status and common risk variants. As reported from detailed studies of the technical issues (39–41), *TMPRSS2:ERG* assessment methods yield very similar results, and we are therefore confident that our results are robust to misclassification. Apart from the detection method, cohort selection is also known to influence the detection rate of *TMPRSS2:ERG* fusions. Of note, the observed *TMPRSS2:ERG* frequency of 56% in the present work is above the consensus of 45–50% reported in literature (reviewed in (42,43)). This might be in part explained by the remarkably different prevalence of *TMPRSS2:ERG* fusions among ethnicities. Studies, which explicitly addressed the population issue, reported 50% or higher *TMPRSS2:ERG* frequencies in subjects of European descent, while significantly less fusions (13%) were observed in non-Europeans (44). Lower *TMPRSS2:ERG* fusion prevalence applied for African Americans (31%) as well as for Asians (16%) (23). The present association study was restricted to European ethnicity, in order to avoid population stratification within the genotype data sets. Therefore, our study only included individuals who have the highest prevalence of *TMPRSS2:ERG* by ethnic origin and in consequence we would expect our *TMPRSS2:ERG* frequencies to reach higher levels as compared with studies with mixed populations.

One important study limitation is the restricted number of cases that had tumor tissue available for somatic typing. Even when phenotyped case groups were compared with a considerable number of controls (n = 7650), power was limited for assessing PrCa risk variants and, thus, some true associations may have been missed. Conversely, the possibility of false-positive results should be considered.

Our finding that known PrCa risk variants at 8q24 and 17q24 are differentially associated with *TMPRSS2:ERG* fusion status further strengthens support for the existence of distinct molecular subtypes in PrCa development. Importantly, this finding should encourage researchers conducting large genetic association studies to ascertain fusion status in order to identify comprehensive sets of subtype-specific risk variants. Recently, genetic epidemiologists have been considering a multifactorial model of PrCa risk, where genotypes of known common variants are converged into polygenic risk scores. Although this approach has promise, the predictive utility of these models is still limited. The knowledge that some risk variants are associated with a particular molecular subtype of PrCa could be incorporated into multifactorial models, thereby refining and improving their ability to identify specific PrCa risk groups.

## Materials and methods

### Study sample

The study samples for each collaborating center are described in detail in the Supplementary Materials. The hypothesis generating discovery sample consisted of PrCa cases and controls genotyped in 2011 using the ‘iCOGS’ array (7), including 27 variants previously shown to influence PrCa risk. Individuals were pre-selected for European ancestry, which was confirmed by principal component analyses of genotyping data. For analyzing the phenotype of interest, the *TMPRSS2:ERG* fusion status, selection criteria for cases were: 1) the availability of primary tumor tissue for *TMPRSS2:ERG* assessment or 2) existing information on fusion status. From five eligible study centers in Finland (TAMPERE), Germany (ULM), the UK (UKGPCS), USA (FHCRC) and Portugal (IPO-PORTO), a total of 552 cases with genotypes (n = 27 variants) and somatic phenotype data were included. An independent sample of cases with available tumor tissue or known *TMPRSS2:ERG* status was used to replicate the results for the five highest ranked candidate variants. The sample comprised 669 cases from Germany (BERLIN and ULM), the UK (UKGPCS), USA (FHCRC) and Portugal (IPO-PORTO). Gene expression analysis of tumor materials was performed using fresh-frozen tissue collections from ULM (35 matched tumor and adjacent benign) and BERLIN (194 specimens, tumor only), and one additional center, ERLANGEN (35 tissue pairs), to increase simple size.

### Genotyping

Genotyping was performed on DNA from peripheral blood lymphocytes. Initially, 27 PrCa risk-associated variants were genotyped by means of the custom Illumina iSelect genotyping array (the iCOGS chip), previously generated by the Collaborative Oncological Gene-Environment Study (COGS). A detailed procedure including genotype calling and quality control has been described earlier in (7).

Genotyping of the replication samples (BERLIN, IPO-PORTO, FHCRC and ULM) was performed using predesigned TaqMan Genotyping Assays for rs1447295, rs16901979, rs10993994, rs1859962 and rs2735839 (Life Technologies, Carlsbad, USA).

### Determination of the TMPRSS2:ERG fusion status

The study groups used FISH or RT-PCR for the assessment of the *TMPRSS2:ERG* fusion status. FISH was applied to FFPE tumor material. Detailed methods of the FISH based *TMPRSS2:ERG* assessment by break apart assays have been described previously for the samples of ULM (14,27), UKGPCS (45), FHCRC (24) and TAMPERE (46). Fresh-frozen material, collected by the BERLIN, ERLANGEN, IPO-PORTO and ULM study groups, was subjected to *TMPRSS2:ERG* detection via RT-PCR using TaqMan primers and probes specific for the most prevalent fusion transcript variant (*T1G4*, *TMPRSS2:ERGa*), which is found in ∼90% of *TMPRSS2:ERG* fusion-positive tumors (41,47). The IPO-PORTO samples were phenotyped as described by Paulo *et al.* (39). Tissues from BERLIN, ERLANGEN and ULM were macro-dissected, followed by RNA isolation using the RNeasy Mini Kit (QIAGEN, Hilden, Germany). The detection of the *TMPRSS2:ERG* fusion transcript was performed using QuantiFast Multiplex RT-PCR +R Kit (QIAGEN, Hilden, Germany) on a VIIA7 Fast Real-Time PCR System (Life Technologies, Carlsbad, USA). Reactions were set up in duplicate in a final volume of 20 µl. Cycling conditions were as follows: 50 °C for 20 min and 95 °C for 5 min for initial reverse transcription and hot start polymerase activation respectively, and subsequently 45 cycles of 94 °C for 15 s and 60 °C for 60 s. Primer and probe sequences are provided in Supplementary Material, Table S4.

### Determination of SOX9 expression

The expression levels of *ALAS1* (reference gene) and *SOX9* were quantified with the QuantiFast Multiplex RT-PCR +R Kit (QIAGEN, Hilden, Germany) on a VIIA7 Fast Real-Time PCR System (Life Technologies, Carlsbad, USA). Reaction and cycling set up is described earlier. The primer and probe sequences are provided in Supplementary Material, Table S4.

### Statistical analysis

Statistical analyses were performed with the Review Manager version 5.1.7 (Copenhagen: The Nordic Cochrane Centre, The Cochrane Collaboration, 2012) and SAS version 9.3.

As heterogeneity between study centers was of interest, we used a meta-analytic approach to assess associations in case-case and case-control comparisons. In detail, for each variant and study center, table-based per-allele odds ratios (ORs) were calculated and Mantel-Haenszel analyses were used to pool the ORs across centers. Fixed effects meta-analyses were preferred over random effect models because the inconsistency of association results across populations (as measured by I^2^) was mostly limited.

Discovery and replication analyses were based on comparisons between *TMPRSS2:ERG* fusion-positive and negative cases (case-case comparisons), where nominal thresholds were applied (*P* = 0.05) based on the limited number of cases available for the analyses. The candidate variant selection for the replication round was also guided by supplementary analysis of the more powerful comparison of case subtypes versus unaffected controls (threshold *P* = 0.00185, according to Bonferroni adjustment for 27 variants). Four variants fulfilled both criteria in the discovery sample (rs2735839, rs10993994, rs16901979 and rs1859962). The candidate variant list was expanded by one further variant (rs1447295) based on the case-case ranking of variants and rankings derived from cancer subtypes vs. controls. Formally, these five variants form the smallest subset of variants ranked ≤ n in case-case comparisons that have also rank ≤ n in cancer subtypes vs. controls. For the combined Mantel-Haenszel analyses of the discovery and replication stages the study wide significance level of *P* = 0.00185 was applied.

The relationship between *TMPRSS2:ERG* fusion status, risk alleles, age at diagnosis and gene fusion detection methods was assessed using multivariable logistic regression, adjusting for study center effects. For this purpose, *TMPRSS2:ERG* status was modeled as the dependent variable, whereas, in addition to center, either age at diagnosis and genotype, or detection method and genotype were included as covariables.

*SOX9* expression levels were calculated by the ΔCt method using *ALAS1* as reference gene, with subsequent log2 transformation to achieve normal distribution of the data for downstream analyses. The comparisons of gene expression between tumor and adjacent benign tissue were performed using the paired t-test. Genotype specific effects on *SOX9* expression levels were tested using a regression model with genotype as an independent variable, adjusted for study center effects. The model was extended for the *TMPRSS2:ERG* status and an interaction term to test for differences between *TMPRSS2:ERG* fusion-positive and negative subsets with regard to the correlation structure of *SOX9* mRNA expression levels and rs1859962 genotypes.

## Supplementary Material

Supplementary Material is available at *HMG* online.

## Supplementary Material

Supplementary Data
