# Supplementary material for: Prostate cancer risk regions at 8q24 and 17q24 are differentially associated with somatic TMPRSS2:ERG fusion status
Source: Hum Mol Genet. 2016 Oct 18;25(24):5490–9. doi: 10.1093/hmg/ddw349 (PMC5418832; doi:10.1093/hmg/ddw349)
Supplement: Supplementary Data [file ddw349_Supp.docx]

**Supplementary Materials**

**Prostate cancer risk regions at 8q24 and 17q24 are differentially associated with somatic *TMPRSS2:ERG* fusion status**

Correspondence should be addressed to Christiane Maier

(christiane.maier@uni-ulm.de)

**p. 2 Supplementary Table S1**

**p. 3 Supplementary Table S2**

**p. 5 Supplementary Table S3**

**p. 6 Supplementary Table S4**

**p. 7 Supplementary Figure S1**

**p. 8 Supplementary Figure S2**

**p. 9 Sample descriptions**

**p. 12 The members of the PRACTICAL consortium**

**p. 13 Extended Acknowledgments**

**p. 14 Reference List**

**Supplementary Table S1:** Case-control comparisons by Mantel-Haenszel analysis for 26 PrCa risk variants using unselected cases, *TMPRSS2:ERG* (*T2E*) phenotyped cases, *T2E* positive and *T2E* negative cases.

| *Variant* | *Region* | *Alleles*  *(non-risk/risk)^a^* | *RAF^b^* | *Color code in Fig 1^c^* | *All cases vs all controls*  *(n = 8,681 vs. n = 7,650)* | | *Phenotyped cases vs controls*  *(n = 552 vs. n = 7,650)* | | *T2E positive cases vs controls*  *(n = 296 vs. n = 7,650)* | | *T2E negative cases vs controls*  *(n = 256 vs. n = 7,650)* | |
| --- | --- | --- | --- | --- | --- | --- | --- | --- | --- | --- | --- | --- |
|  |  |  |  |  | *OR [95% CI]* | *p-value* | *OR [95% CI]* | *p-value* | *OR [95% CI]* | *p-value* | *OR [95% CI]* | *p-value* |
| rs1447295 | 8q24 | C/A | 0.12 |  | 1.45 [1.36-1.55] | **3.6 × 10^-31^** | 1.51 [1.27-1.78] | **2.0 × 10^-06^** | 1.33 [1.05-1.67] | 0.0164 | 1.75 [1.39-2.21] | **2.0 × 10^-06^** |
| rs10993994 | 10q11 | C/T | 0.37 |  | 1.28 [1.22-1.33] | **1.5 × 10^-26^** | 1.28 [1.13-1.45] | **1.4 × 10^-04^** | 1.47 [1.24-1.74] | **6.5 × 10^-06^** | 1.09 [0.91-1.31] | 0.3678 |
| rs1512268 | 8p21.2 | G/A | 0.42 |  | 1.26 [1.20-1.32] | **9.2 × 10^-25^** | 1.27 [1.12-1.44] | **1.7 × 10^-04^** | 1.22 [1.03-1.44] | 0.0190 | 1.34 [1.12-1.60] | **0.0014** |
| rs16901979 | 8q24 | C/A | 0.03 |  | 1.71 [1.53-1.92] | **4.9 × 10^-21^** | 1.72 [1.29-2.29] | **2.0 × 10^-04^** | 1.17 [0.75-1.83] | 0.4751 | 2.37 [1.66-3.39] | **1.2 × 10^-06^** |
| rs10486567 | 7p15 | A/G | 0.75 |  | 1.18 [1.12-1.24] | **8.7 × 10^-10^** | 1.34 [1.15-1.57] | **2.2 × 10^-04^** | 1.25 [1.02-1.53] | 0.0328 | 1.46 [1.16-1.84] | **0.0011** |
| rs1859962 | 17q24 | T/G | 0.47 |  | 1.21 [1.16-1.27] | **2.4 × 10^-18^** | 1.25 [1.10-1.42] | **5.4 × 10^-04^** | 1.40 [1.19-1.66] | **7.6 × 10^-05^** | 1.09 [0.91-1.30] | 0.3396 |
| rs6465657 | 7q21 | T/C | 0.47 |  | 1.16 [1.11-1.21] | **2.1 × 10^-11^** | 1.21 [1.06-1.37] | 0.0034 | 1.32 [1.11-1.56] | **0.0013** | 1.09 [0.91-1.30] | 0.3588 |
| rs6983267 | 8q24 | T/G | 0.51 |  | 1.30 [1.24-1.35] | **4.8 × 10^-31^** | 1.20 [1.06-1.37] | 0.0039 | 1.23 [1.04-1.46] | 0.0150 | 1.17 [0.98-1.40] | 0.0853 |
| rs2735839 | 19q13 | A/G | 0.85 |  | 1.34 [1.26-1.43] | **8.7 × 10^-20^** | 1.32 [1.09-1.60] | 0.0040 | 1.73 [1.31-2.30] | **1.2 × 10^-04^** | 1.02 [0.79-1.31] | 0.8930 |
| rs7931342 | 11q13 | T/G | 0.49 |  | 1.20 [1.15-1.26] | **1.8 × 10^-16^** | 1.17 [1.03-1.33] | 0.0135 | 1.09 [0.93-1.29] | 0.2925 | 1.28 [1.07-1.53] | 0.0071 |
| rs7679673 | 4q24 | A/C | 0.58 |  | 1.13 [1.08-1.18] | **1.0 × 10^-07^** | 1.12 [0.99-1.28] | 0.0751 | 1.12 [0.95-1.33] | 0.1807 | 1.13 [0.94-1.35] | 0.2048 |
| rs9364554 | 6q25 | C/T | 0.29 |  | 1.12 [1.07-1.18] | **2.2 × 10^-06^** | 1.13 [0.99-1.30] | 0.0769 | 1.22 [1.02-1.46] | 0.0284 | 1.04 [0.85-1.27] | 0.7031 |
| rs721048 | 2p15 | G/A | 0.18 |  | 1.10 [1.04-1.16] | **0.0013** | 1.13 [0.96-1.33] | 0.1307 | 1.15 [0.93-1.42] | 0.1902 | 1.11 [0.88-1.39] | 0.3802 |
| rs5945619 | Xp11 | T/C | 0.38 |  | 1.24 [1.16-1.32] | **3.7 × 10^-11^** | 1.14 [0.95-1.37] | 0.1484 | 1.15 [0.90-1.46] | 0.2593 | 1.14 [0.88-1.48] | 0.3130 |
| rs17021918 | 4q22.3 | T/C | 0.65 |  | 1.12 [1.07-1.18] | **1.0 × 10^-06^** | 1.10 [0.96-1.26] | 0.1706 | 1.21 [1.01-1.45] | 0.0368 | 0.98 [0.82-1.19] | 0.8600 |
| rs11649743 | 17q12 | A/G | 0.82 |  | 1.13 [1.07-1.20] | **3.0 × 10^-05^** | 1.12 [0.95-1.33] | 0.1767 | 1.28 [1.01-1.62] | 0.0378 | 0.97 [0.77-1.22] | 0.7935 |
| rs6763931 | 3q23 | C/T | 0.44 |  | 1.07 [1.02-1.11] | 0.0046 | 1.07 [0.95-1.22] | 0.2679 | 1.07 [0.91-1.27] | 0.4045 | 1.08 [0.90-1.29] | 0.4090 |
| rs2928679 | 8p21.2 | C/T | 0.42 |  | 1.09 [1.04-1.13] | **3.1 × 10^-04^** | 1.07 [0.94-1.21] | 0.3201 | 1.15 [0.97-1.37] | 0.0996 | 0.98 [0.82-1.18] | 0.8316 |
| rs130067 | 6p21 | T/G | 0.20 |  | 1.07 [1.02-1.13] | 0.9142 | 0.95 [0.81-1.11] | 0.5188 | 0.97 [0.79-1.20] | 0.8038 | 0.92 [0.74-1.16] | 0.4905 |
| rs5919432 | Xq12 | G/A | 0.81 |  | 1.13 [1.04-1.22] | 0.0032 | 1.07 [0.85-1.35] | 0.5575 | 1.11 [0.82-1.52] | 0.4981 | 1.04 [0.75-1.44] | 0.8084 |
| rs10875943 | 12q13 | T/C | 0.30 |  | 1.10 [1.05-1.15] | **1.2 × 10^-04^** | 1.04 [0.90-1.19] | 0.6199 | 0.98 [0.82-1.18] | 0.8666 | 1.10 [0.91-1.34] | 0.3132 |
| rs12621278 | 2q31.1 | G/A | 0.94 |  | 1.34 [1.22-1.48] | **6.0 × 10^-09^** | 1.07 [0.80-1.43] | 0.6558 | 1.09 [0.74-1.62] | 0.6622 | 1.03 [0.68-1.54] | 0.9045 |
| rs1465618 | 2p21 | G/A | 0.21 |  | 1.10 [1.04-1.16] | **3.6 × 10^-04^** | 1.03 [0.89-1.20] | 0.6832 | 1.08 [0.89-1.32] | 0.4293 | 0.99 [0.79-1.23] | 0.9075 |
| rs4962416 | 10q26 | T/C | 0.25 |  | 1.06 [1.01-1.11] | 0.0271 | 0.99 [0.85-1.14] | 0.8508 | 1.05 [0.87-1.27] | 0.6202 | 0.92 [0.74-1.13] | 0.4328 |
| rs2660753 | 3p12 | C/T | 0.10 |  | 1.06 [0.99-1.14] | 0.1156 | 0.99 [0.80-1.22] | 0.9082 | 1.21 [0.93-1.57] | 0.1576 | 0.76 [0.55-1.06] | 0.1051 |
| rs12500426 | 4q22.3 | C/A | 0.47 |  | 1.06 [1.01-1.11] | 0.0118 | 1.00 [0.88-1.13] | 0.9920 | 1.01 [0.85-1.19] | 0.9477 | 0.99 [0.83-1.19] | 0.9146 |

^a^ Risk alleles for prostate cancer

^b^ Risk allele frequencies in 7,650 controls

^c^ Color code presented in Figure 1: Black circles represent variants significant after Bonferroni correction for multiple testing (threshold p = 0.00185), while gray and open circles represent variants that did and did not reach nominal significance, respectively.

**Supplementary Table S2:** Overview of the 27 analyzed PrCa risk variants from the discovery dataset, association results of the *TMPRSS2:ERG* (*T2E*) positive versus *T2E* negative (case-case) comparisons and the corresponding ranks of the case-case and *T2E* subtype vs. controls comparisons (see Supplementary Table S1).

|  |  | |  | *Risk allele frequency^b^* | | |  | *Heterogeneity* | |  | *T2E positive vs T2E negative (case-case comparison)^c^* | |  |  | |
| --- | --- | --- | --- | --- | --- | --- | --- | --- | --- | --- | --- | --- | --- | --- | --- |
| *Variant* | *Region* | | *Alleles*  *(non-risk/risk)^a^* | *Cases* | *T2E +* | *T2E -* |  | *I^2^[%]* | *p-value* |  | *OR^d^ [95% CI]* | *p-value* |  | *Rank in*  *case-case comparison* | *Rank in T2E subtype vs controls* |
| rs2735839 | | 19q13 | A/G | 0.88 | 0.91 | 0.85 |  | 0 | 0.53 |  | 1.73 [1.20 - 2.51] | 0.0035 |  | 1 | 5 |
| rs10993994 | | 10q11 | C/T | 0.43 | 0.46 | 0.39 |  | 0 | 0.45 |  | 1.35 [1.06 - 1.72] | 0.0151 |  | 2 | 3 |
| rs16901979 | | 8q24 (R2) | C/A | 0.05 | 0.04 | 0.07 |  | 0 | 0.62 |  | 0.53 [0.31 - 0.91] | 0.0214 |  | 3 | 1 |
| rs1859962 | | 17q24 | T/G | 0.52 | 0.56 | 0.50 |  | 19 | 0.29 |  | 1.29 [1.01 - 1.64] | 0.0375 |  | 4 | 4 |
| rs1447295 | | 8q24 (R1) | C/A | 0.17 | 0.16 | 0.19 |  | 31 | 0.21 |  | 0.76 [0.56 - 1.04] | 0.0891 |  | 5 | 2 |
| rs11649743 | | 17q12 | A/G | 0.83 | 0.85 | 0.81 |  | 4 | 0.38 |  | 1.31 [0.96 - 1.80] | 0.0930 |  | 6 | 13 |
| rs17021918 | | 4q22.3 | T/C | 0.68 | 0.69 | 0.65 |  | 65 | 0.02 |  | 1.19 [0.92 - 1.53] | 0.1770 |  | 7 | 12 |
| rs2660753 | | 3p12 | C/T | 0.10 | 0.12 | 0.10 |  | 0 | 0.44 |  | 1.30 [0.88 - 1.92] | 0.1936 |  | 8 | 15 |
| rs10486567 | | 7p15 | A/G | 0.78 | 0.79 | 0.82 |  | 34 | 0.19 |  | 0.83 [0.61 - 1.11] | 0.2113 |  | 9 | 6 |
| rs7931342 | | 11q13 | T/G | 0.54 | 0.51 | 0.55 |  | 0 | 0.75 |  | 0.86 [0.68 - 1.10] | 0.2225 |  | 10 | 9 |
| rs9364554 | | 6q25 | C/T | 0.31 | 0.32 | 0.30 |  | 26 | 0.25 |  | 1.17 [0.90 - 1.52] | 0.2301 |  | 11 | 11 |
| rs4962416 | | 10q26 | T/C | 0.27 | 0.25 | 0.24 |  | 0 | 0.92 |  | 1.13 [0.86 - 1.49] | 0.3843 |  | 12 | 22 |
| rs10875943 | | 12q13 | T/C | 0.32 | 0.30 | 0.32 |  | 0 | 0.82 |  | 0.89 [0.69 - 1.16] | 0.3898 |  | 13 | 19 |
| rs1512268 | | 8p21.2 | G/A | 0.48 | 0.48 | 0.50 |  | 0 | 0.87 |  | 0.91 [0.72 - 1.16] | 0.4533 |  | 14 | 8 |
| rs2928679 | | 8p21.2 | C/T | 0.44 | 0.45 | 0.44 |  | 49 | 0.09 |  | 1.07 [0.84 - 1.37] | 0.5687 |  | 15 | 14 |
| rs1465618 | | 2p21 | G/A | 0.23 | 0.23 | 0.21 |  | 59 | 0.05 |  | 1.08 [0.81 - 1.44] | 0.6171 |  | 16 | 21 |
| rs6983267 | | 8q24 (R3) | T/G | 0.57 | 0.56 | 0.55 |  | 0 | 0.79 |  | 1.05 [0.83 - 1.33] | 0.6965 |  | 17 | 10 |
| rs6763931 | | 3q23 | C/T | 0.45 | 0.46 | 0.45 |  | 0 | 0.45 |  | 1.04 [0.82 - 1.32] | 0.7339 |  | 18 | 20 |
| rs5919432 | | Xq12 | G/A | 0.83 | 0.82 | 0.82 |  | 67 | 0.02 |  | 1.07 [0.70 - 1.65] | 0.7490 |  | 19 | 24 |
| rs12621278 | | 2q31.1 | G/A | 0.96 | 0.95 | 0.95 |  | 4 | 0.38 |  | 1.07 [0.62 - 1.85] | 0.8103 |  | 20 | 25 |
| rs12500426 | | 4q22.3 | C/A | 0.49 | 0.48 | 0.47 |  | 0 | 0.60 |  | 0.97 [0.76 - 1.24] | 0.8259 |  | 21 | 26 |
| rs130067 | | 6p21 | T/G | 0.21 | 0.20 | 0.19 |  | 0 | 0.77 |  | 1.03 [0.77 - 1.40] | 0.8259 |  | 22 | 23 |
| rs7679673 | | 4q24 | A/C | 0.61 | 0.60 | 0.61 |  | 15 | 0.32 |  | 0.98 [0.77 - 1.25] | 0.8572 |  | 23 | 16 |
| rs721048 | | 2p15 | G/A | 0.19 | 0.19 | 0.20 |  | 8 | 0.36 |  | 1.02 [0.75 - 1.38] | 0.8966 |  | 24 | 17 |
| rs6465657 | | 7q21 | T/C | 0.51 | 0.49 | 0.48 |  | 52 | 0.08 |  | 1.02 [0.80 - 1.29] | 0.8966 |  | 25 | 7 |
| rs5945619 | | Xp11.22 | T/C | 0.43 | 0.42 | 0.41 |  | 0 | 0.59 |  | 1.01 [0.72 - 1.43] | 0.9442 |  | 26 | 18 |
| rs7127900^e^ | | 11p15.5 | G/A | 0.23 | 0.27 | 0.26 |  | 71 | 0.007 |  | 1.06 [0.81 - 1.39] | 0.6527 |  | omitted | omitted |

^a^ Risk alleles for prostate cancer

^b^ Calculated for unselected cases (n = 8,681) and *T2E* phenotyped cases (positive, n = 296; negative, n = 256) of the discovery dataset

^c^ Fixed-effects meta-analysis of five sample sets FHCRC I, TAMPERE, IPO-PORTO I, UKGPCS and ULM I with a total number of 296 *TMRSS2:ERG* fusion-positive and 256 *TMPRSS2:ERG* fusion-negative prostate cancer cases

^d^ Odds ratios less than 1 imply an overrepresentation of PrCa risk alleles in *TMPRSS2:ERG* negative cases, whereas odds ratios above 1 indicate an overrepresentation in *TMPRSS2:ERG* positive cases

^e^ Not considered due to sampling bias

**Supplementary Table S3:** Multivariable logistic regression analyses for testing potential confounders of associations with *TMPRSS2:ERG* (*T2E*) fusion status. Risk variants, age at diagnosis and *T2E* detection methods are included as covariables.

| *Parameters* | *OR [95% CI]* | *p-value* |
| --- | --- | --- |
| Age at diagnosis association with *T2E* |  |  |
| Crude OR (per year) | 0.96 [0.95-0.98] | 4.7 × 10^-05^ |
| Adjusted for rs16901979 (per year) | 0.96 [0.95-0.98] | 3.3 × 10^-05^ |
| Adjusted for rs1859962 (per year) | 0.96 [0.95-0.98] | 3.6 × 10^-05^ |
|  |  |  |
| rs16901979 association with *T2E* |  |  |
| Crude OR (per-allele) | 0.54 [0.37-0.77] | 0.0008 |
| Adjusted for age (per-allele) | 0.52 [0.36-0.75] | 0.0005 |
| Adjusted for method (per-allele) | 0.54 [0.37-0.77] | 0.0008 |
|  |  |  |
| rs1859962 association with *T2E* |  |  |
| Crude OR (per-allele) | 1.31 [1.11-1.54] | 0.0014 |
| Adjusted for age (per-allele) | 1.31 [1.11-1.55] | 0.0014 |
| Adjusted for method (per-allele) | 1.31 [1.11-1.54] | 0.0013 |

**Supplementary Table S4:** Primer and probe sequences for the qPCR based detection of the most prevalent *TMPRSS2:ERG* fusion transcript *T1G4* (*TMPRSS2-ERGa*) and the determination of *SOX9* expression levels. For the latter, *ALAS1* served as reference gene. Primers were obtained from biomers.net (Ulm, Germany), TaqMan MGB probes from Life Technologies (Carlsbad, USA), respectively.

| **qPCR Primer** |  |  |
| --- | --- | --- |
| Oligo name |  | Sequence 5' - 3' |
| f_ALAS1 |  | TGATGAACTACTTCCTTGAGAATC |
| r_ALAS1 |  | GAATGAGGCTTCAGTTCCA |
| f_SOX9 |  | CACTTGCACAACGCCGA |
| r_SOX9 |  | TCGCTCTCGTTCAGAAGTCTC |
| f_T1G4 |  | TGGAGCGCGGCAGGAAG |
| r_T1G4 |  | TCCGTAGGCACACTCAAACA |

|  |  |  |
| --- | --- | --- |
| **TaqMan MGB Probes** |  |  |
| Probe name |  | Dye Sequence 5' - 3' Quencher |
| p_ALAS1 |  | VIC CTAGTCACATGGAAGCAA MGB-NFQ |
| p_SOX9 |  | 6FAM CAAGACGCTGGGCAA MGB-NFQ |
| p_T1G4 |  | 6FAM TCCTCACTCACAACTGATAA MGB-NFQ |





**Supplementary Figure S1:** Forest plots of five PrCa risk variants evaluated for subtype preference in the discovery and the independent replication datasets, as well as the combined analyses. The meta-analysis compared *TMPRSS2:ERG* (*T2E*) fusion-positive versus fusion-negative PrCa cases using Mantel-Haenszel analysis with fixed-effects. For each study, the position of the squares represent the per allele odds ratio (OR), square size correspond to study weight and the lines denote the 95% confidence intervals (CI). The diamonds indicate the combined OR including 95% confidence intervals of the subgroup analyses. ORs less than 1 (rs16901979 and rs1447295 on 8q24) imply an overrepresentation of the PrCa risk allele in *T2E* fusion-negative cases, while ORs above 1 indicate an overrepresentation in fusion-positive cases. Study heterogeneity I^2^ and the corresponding p-value are presented in the lower panel.


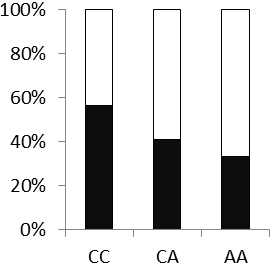


*n* = 898

*n* = 98

*n* = 3

8q24, rs16901979

*T2E* freq

56,6%

40,8%

33,3%


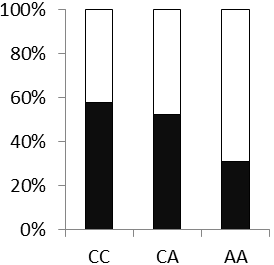


*n* = 905

*n* = 279

*n* = 29

8q24, rs1447295

*T2E* freq

57,7%

52,3%

31,0%


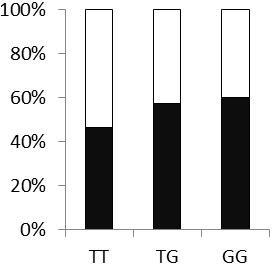


*n* = 255

*n* = 605

*n* = 358

17q24, rs1859962

*T2E* freq

46,3%

57,5%

60,1%


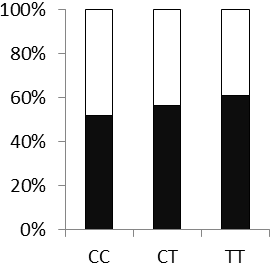


*n* = 388

*n* = 575

*n* = 255

10q11, rs10993994

52,1%

56,5%

60,8%

*T2E* freq


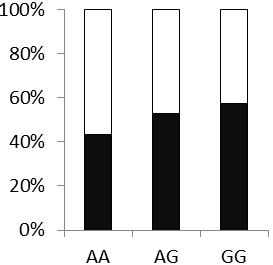


*n* = 23

*n* = 296

*n* = 902

19q13, rs2735839

43,5%

52,7%

57,4%

*T2E* freq

**Supplementary Figure S2:** Frequency of *TMPRSS2:ERG* (*T2E*) positive tumors among subjects stratified by genotypes. Samples consist of all 1,221 phenotyped cases included in the discovery and the replication dataset. Variants associated with fusion-negative PrCa show a decline of the *T2E* frequency with number of risk alleles (rs16901979, p-trend = 0.0022; rs1447295, p-trend = 0.0052), whereas candidates for fusion-positive PrCa tend towards an increase of *T2E* frequency with number of risk alleles (rs1859962, p-trend = 0.0012; rs10993994 p-trend = 0.027; rs2735839, p-trend = 0.066). The overall frequency of *T2E* positive cases in the total data set is 56%.

**Sample descriptions**

**Prostate cancer samples**

All groups except BERLIN and ERLANGEN are members of the PRACTICAL (Prostate Cancer Association Group to Investigate Cancer Associated Alterations in the Genome) consortium. All studies have been approved by their respective local institutional review board. The study samples are described below. For the present study, we only selected individuals with European ancestry.

**BERLIN: Department of Urology, University Hospital Charité, Berlin, Germany**

For this study, total RNA of 198 prostate cancer tissues as well as lymphocyte DNA from patients who underwent radical prostatectomy between 2000 and 2006 at Charité - Universitätsmedizin, Klinikum Benjamin Franklin was obtained by standard isolation procedures. All patients gave their informed consent in accordance with guidelines provided by the local Ethics Review Board. The RNA was used for *TMPRSS2:ERG* detection as well as for genotype specific expression analysis of the candidate genes *MYC* and *SOX9*.

**ERLANGEN: Friedrich-Alexander University of Erlangen-Nürnberg, Erlangen, Germany**

A total of 35 matched pairs of fresh-frozen tumor and adjacent benign tissue was retrieved from the tissue bio-repository of the Comprehensive Cancer Center of the University Hospital Erlangen. Patients underwent radical prostatectomy between 2008 and 2010. After review by an experienced pathologist, DNA and RNA was extracted by standardized isolation procedures using Trizol reagent. RNA was used for expression profiling of *MYC* and *SOX9*. The *TMPRSS2:ERG* fusion status of this sample was determined by qPCR and genotypes were derived from DNA of benign tissue.

**FHCRC: Fred Hutchinson Cancer Research Center, Seattle, USA**

The study population consists of participants from two population-based case-control studies in Caucasian residents of King County, Washington (Study I and Study II), which have been previously described (1,2). Incident cases with histologically confirmed prostate cancer were ascertained from the Seattle-Puget Sound Surveillance, Epidemiology and End Results cancer registry. In Study I, cases were diagnosed between January 1, 1993, and December 31, 1996 and were 40-64 years of age at diagnosis. In Study II, cases were diagnosed between January 1, 2002, and December 31, 2005 and were 35-74 years of age at diagnosis. A comparison group of controls without a history of prostate cancer, residing in King County, Washington, was identified for each study using random digit telephone dialing. Controls were frequency-matched to cases by five-year age groups and recruited evenly throughout each ascertainment period for cases.

For the current study, genotype data from the iCOGS array was available for 637 controls and 604 cases. 174 of these cases were phenotyped for the *TMPRSS2:ERG* fusion. Further 218 independent PrCa cases with *TMPRSS2:ERG* data available were included in the replication set.

**IPO-PORTO: Instituto Português de Oncologia do Porto, Porto, Portugal**

The IPO-Porto prostate cancer study includes patients with clinically localized prostate adenocarcinoma consecutively diagnosed and treated with open radical prostatectomy at the Portuguese Oncology Institute - Porto, Portugal, since 1999. The project involves sample collection of peripheral blood, urine and fresh-frozen tumor tissue. Relevant clinical data, namely Gleason grading, clinico-pathological staging and PSA level at diagnosis, were obtained from medical records.

For the current study, genotype data from the iCOGS study was available for 66 controls and 183 cases. 18 of these cases were phenotyped for the *TMPRSS2:ERG* fusion. Further 146 independent PrCa cases with *TMPRSS2:ERG* data available, were included in the replication approach.

**TAMPERE:** **Institute of Biomedical Technology/BioMediTech and Fimlab Laboratories, University of Tampere, Tampere, Finland**

The first set of unselected cases and controls (PSA < 4 μg/ml) were collected in Tampere, Finland and all are of Finnish origin. The mean age of diagnosis was 68.7 years (range 36-94). The patients were diagnosed with prostate cancer from 1993-2008 at the Tampere University Hospital, Department of Urology. Tampere University Hospital is a regional referral center in the area for all patients with prostate cancer, which results in an unselected, population-based collection of patients. The other unselected set of cases and controls was collected in the Finnish arm of The European Randomized Study of Screening for Prostate Cancer, which was initiated in the early 1990s to evaluate the effect of screening with PSA testing on death rates from prostate cancer. These men were born in years 1933, 1937 and 1941 and were randomly assigned to a group that was offered PSA screening at an average of once every 4 years or to a control group that did not receive such screening. In addition to these two sporadic sample sets, familial cancer cases (mean age at diagnosis 70 years) from Finnish prostate cancer families were genotyped.

For the current study, genotype data from the iCOGS study was available for 2413 controls and 2754 cases. 174 of these cases were phenotyped for the *TMPRSS2:ERG* fusion.

**UKGPCS: UK Genetic Prostate Cancer Study, The Institute of Cancer Research, Sutton, UK**

DNA from blood was collected from prostate cancer patients throughout the UK, aged ≤ 60 years at diagnosis, and from a systematic series of cases from the prostate cancer clinic at The Royal Marsden NHS Foundation Trust. Diagnosis was confirmed from medical record or death certificate and 60% of tumors were detected clinically.

For the current study, genotype data from the iCOGS study was available for 4180 controls and 4549 cases. 129 of these cases were phenotyped for the *TMPRSS2:ERG* fusion.

**ULM: Institute of Human Genetics and Department of Urology, University of Ulm, Ulm, Germany**

Cases were recruited in two different ways. Familial prostate cancer probands (index cases) were ascertained from all over Germany from 1997-2007. They were advised by their attending physicians to contact the Clinic of Urology of Ulm. The positive family history was then verified by reviewing medical records or death certificates of family members. Only one member of each family (e.g. the index proband) was enrolled in the present study. Sporadic cases, who reported no relatives affected with prostate cancer, were collected in Ulm during their course of treatment (e.g. radical prostatectomy) at the Department for Urology of the University Hospital Ulm. The control group consists of age-matched healthy men and male population controls of unknown disease status.

For the current study, genotype data from the iCOGS study was available for 354 controls (209 age matched, 145 unselected males) and for 591 cases. 57 of these cases were phenotyped for the *TMPRSS2:ERG* fusion. Further 107 independent PrCa cases with *TMPRSS2:ERG* data available, were included in the replication approach.

Pairs of fresh frozen tumor and adjacent benign tissue of 35 independent PrCa cases were used for expression analysis of the candidate genes *MYC* and *SOX9*.

**The PRACTICAL Consortium:**

Rosalind Eeles ^1, 2^, Doug Easton ^3^, Kenneth Muir ^4^, Graham Giles ^5, 6^, Fredrik Wiklund ^7^, Henrik Gronberg ^7^, Christopher Haiman ^8^, Johanna Schleutker ^9, 10^, Maren Weischer ^11^, Ruth C. Travis ^12^, David Neal ^13^, Paul Pharoah ^14^, Kay-Tee Khaw ^15^, Janet L. Stanford ^16, 17^, William J. Blot ^18^, Stephen Thibodeau ^19^, Christiane Maier ^20, 21^, Adam S. Kibel ^22, 23^, Cezary Cybulski ^24^, Lisa Cannon-Albright ^25^, Hermann Brenner ^26^, Jong Park ^27^, Radka Kaneva ^2^8, Jyotnsa Batra ^29^, Manuel R. Teixeira ^30^, Zsofia Kote-Jarai ^1^, Ali Amin Al Olama ^3^, Sara Benlloch ^3^

^1^ The Institute of Cancer Research, 15 Cotswold Road, Sutton, Surrey, SM2 5NG, UK, ^2^ Royal Marsden NHS Foundation Trust, Fulham and Sutton, London and Surrey, UK, ^3^ Centre for Cancer Genetic Epidemiology, Department of Public Health and Primary Care, University of Cambridge, Strangeways Laboratory, Worts Causeway, Cambridge, UK, ^4^ University of Warwick, Coventry, UK, ^5^ Cancer Epidemiology Centre, The Cancer Council Victoria, 1 Rathdowne street, Carlton Victoria, Australia, ^6^ Centre for Molecular, Environmental, Genetic and Analytic Epidemiology, The University of Melbourne, Victoria, Australia, ^7^ Department of Medical Epidemiology and Biostatistics, Karolinska Institute, Stockholm, Sweden, ^8^ Department of Preventive Medicine, Keck School of Medicine, University of Southern California/Norris Comprehensive Cancer Center, Los Angeles, California, USA, ^9^ Department of Medical Biochemistry and Genetics, University of Turku, Turku, Finland, ^10^ Institute of Biomedical Technology/BioMediTech, University of Tampere and FimLab Laboratories, Tampere, Finland, ^11^ Department of Clinical Biochemistry, Herlev Hospital, Copenhagen University Hospital, Herlev Ringvej 75, DK-2730 Herlev, Denmark, ^12^ Cancer Epidemiology Unit, Nuffield Department of Clinical Medicine, University of Oxford, Oxford, UK, ^13^ Surgical Oncology (Uro-Oncology: S4), University of Cambridge, Box 279, Addenbrooke’s Hospital, Hills Road, Cambridge, UK and Cancer Research UK Cambridge Research Institute, Li Ka Shing Centre, Cambridge, UK, ^14^ Centre for Cancer Genetic Epidemiology, Department of Oncology, University of Cambridge, Strangeways Laboratory, Worts Causeway, Cambridge, UK, ^15^ Cambridge Institute of Public Health, University of Cambridge, Forvie Site, Robinson Way, Cambridge CB2 0SR, ^16^ Division of Public Health Sciences, Fred Hutchinson Cancer Research Center, Seattle, Washington, USA, ^17^ Department of Epidemiology, School of Public Health, University of Washington, Seattle, Washington, USA, ^18^ International Epidemiology Institute, 1455 Research Blvd., Suite 550, Rockville, MD 20850, ^19^ Mayo Clinic, Rochester, Minnesota, USA, ^20^ Institute of Human Genetics University Hospital Ulm, Germany, ^21^ Department of Urology, University Hospital Ulm, Germany, ^22^ Brigham and Women's Hospital/Dana-Farber Cancer Institute, 45 Francis Street- ASB II-3, Boston, MA 02115, ^23^ Washington University, St Louis, Missouri, ^24^ International Hereditary Cancer Center, Department of Genetics and Pathology, Pomeranian Medical University, Szczecin, Poland, ^25^ Division of Genetic Epidemiology, Department of Medicine, University of Utah School of Medicine, ^26^ Division of Clinical Epidemiology and Aging Research, German Cancer Research Center, Heidelberg Germany, ^27^ Division of Cancer Prevention and Control, H. Lee Moffitt Cancer Center, 12902 Magnolia Dr., Tampa, Florida, USA, ^28^ Molecular Medicine Center and Department of Medical Chemistry and Biochemistry, Medical University - Sofia, 2 Zdrave St, 1431, Sofia, Bulgaria, ^29^ Australian Prostate Cancer Research Centre-Qld, Institute of Health and Biomedical Innovation and Schools of Life Science and Public Health, Queensland University of Technology, Brisbane, Australia, ^30^ Department of Genetics, Portuguese Oncology Institute, Porto, Portugal and Biomedical Sciences Institute (ICBAS), Porto University, Porto, Portugal

**Extended Acknowledgment**

The Genotype-Tissue Expression (GTEx) Project was supported by the Common Fund of the Office of the Director of the National Institutes of Health. Additional funds were provided by the NCI, NHGRI, NHLBI, NIDA, NIMH, and NINDS. Donors were enrolled at Biospecimen Source Sites funded by NCI\SAIC-Frederick, Inc. (SAIC-F) subcontracts to the National Disease Research Interchange (10XS170), Roswell Park Cancer Institute (10XS171), and Science Care, Inc. (X10S172). The Laboratory, Data Analysis, and Coordinating Center (LDACC) was funded through a contract (HHSN268201000029C) to The Broad Institute, Inc. Biorepository operations were funded through an SAIC-F subcontract to Van Andel Institute (10ST1035). Additional data repository and project management were provided by SAIC-F (HHSN261200800001E). The Brain Bank was supported by supplements to University of Miami grants DA006227 & DA033684 and to contract N01MH000028. Statistical Methods development grants were made to the University of Geneva (MH090941 & MH101814), the University of Chicago (MH090951, MH090937, MH101820, MH101825), the University of North Carolina - Chapel Hill (MH090936 & MH101819), Harvard University (MH090948), Stanford University (MH101782), Washington University St Louis (MH101810), and the University of Pennsylvania (MH101822). The data used for the analyses described in this manuscript were obtained from the GTEx Portal and dbGaP accession number phs000424.v6.p1 on 09/16/2016.

**Reference List**

1. Agalliu,I., Salinas,C.A., Hansten,P.D., Ostrander,E.A., Stanford,J.L. (2008) Statin use and risk of prostate cancer: results from a population-based epidemiologic study. *Am. J. Epidemiol.*, **168**, 250-260.

2. Stanford,J.L., Wicklund,K.G., McKnight,B., Daling,J.R., Brawer,M.K. (1999) Vasectomy and risk of prostate cancer. *Cancer Epidemiol. Biomarkers Prev.*, **8**, 881-886.
